# Results and complications of bilateral limb lengthening in achondroplasia: a retrospective analysis

**DOI:** 10.3389/fped.2023.1281099

**Published:** 2023-11-03

**Authors:** Fabio Verdoni, Riccardo Giorgino, Camilla Virgilio, Alessandra Nannini, Marco Viganò, Domenico Curci, Giuseppe Michele Peretti, Laura Mangiavini

**Affiliations:** ^1^ IRCCS Istituto Ortopedico Galeazzi, Milan, Italy; ^2^ Residency Program in Orthopedics and Traumatology, University of Milan, Milan, Italy; ^3^ Faculty of Medicine and Surgery, University of Milan, Milan, Italy; ^4^ Department of Biomedical Sciences for Health, University of Milan, Milan, Italy

**Keywords:** limb lengthening, achondroplasia, external fixation, outcomes, complications, tibia, femur

## Abstract

**Background:**

Achondroplasia is one of the main causes of disharmonic dwarfism. Patients with achondroplasia might have physical and psychological limitations due to their disproportionate stature. Surgical limb lengthening is the only practical option available to achieve a stature comparable to normal population range. The purpose of this study is to analyze results and complications of our lengthening protocol.

**Methods:**

A retrospective analysis was performed on 33 patients with achondroplasia (21 females and 12 males) undergoing simultaneous bilateral tibia or femur lengthening in four surgical stages from 2017 to 2021 (46 lengthening procedures, with a total of 56 tibias and 36 femurs). For each patient, patients’ characteristics and antero-posterior and lateral radiographs were obtained. The following parameters were analyzed: duration of lengthening with external fixator, amount of lengthening, complications or events that influenced outcomes and the healing index (HI).

**Results:**

The average tibial and femoral gain was 7.9 cm and 6.9 cm, respectively. The tibiae achieved better results than the femurs (*p* = 0.005). Nineteen complications were reported for 92 segments (20.7%), and the variables influencing complications were: step (*p* = 0.002) and fixation duration (*p* = 0.061).

**Conclusions:**

Bilateral parallel lower limb lengthening in four surgical steps may be a viable technique in patients with achondroplasia.

## 1. Introduction

Achondroplasia is the most common form of skeletal dysplasia and represents one of the main causes of disharmonic dwarfism (1, 2). The cause of the disease lies in a mutation of the FGFR3 (Fibroblast Growth Factor Receptor 3) gene, which is expressed at the level of growth cartilage (1, 3). The clinical phenotype is characterized by disproportionately reduced stature with rhizomelia, an average-size trunk, a disproportionately large head, a prominent forehead, and a flattened bridge of the nose (1). Mild to moderate hypotonia might be present at birth, usually due to a spinal cord compression in the cervical spine. This condition can cause a retardation in motor skills development, while cognitive development is normal (2, 4). In the first year of life growth rate is similar to non-affected children; in the first decade, it drops to the 3rd percentile, while it can increase again during puberty. The average height in adult age is 131 ± 5.6 cm in men and 124 ± 5.9 cm in women (4). Patients with achondroplasia might have physical and psychological limitations due to the disproportionate stature of the body and difficulties in daily life activities (5).

In addition to drug therapy, surgical limb lengthening is one of the possible elongation procedures and represents a challenging pathway burdened by a high rate of complications. However, this procedure remains the only practical option for patients with achondroplasia to achieve a stature comparable to the normal population range at maturity and restore body proportions more similar to the normal population (6, 7). The purpose of this therapy is not merely cosmetic but primarily functional; with hypometric limb lengthening, it is also possible to correct associated axial deviations and prevent joint degeneration. It also enables patients to gain greater autonomy in daily life activities, such as personal hygiene, use of public transportation, and sports, all while improving their psychological and emotional state (8). The biological principle at the base of surgical limb lengthening is gradual and constant tissue traction. This strategy can activate proliferative and biosynthetic functions. After osteotomy and an external fixator (EF) placement, ex-novo bone production begins. It is called “distraction osteogenesis” and consists of three different stages: latency phase, distraction (usually 1 mm/day), and consolidation (9).

Moreover, lengthening protocols used in achondroplasia are numerous. However, there need to be more specific and universal guidelines regarding the patient's age at the beginning of surgery, the sequence of steps, the surgical technique, and the length gain for each bone segment (10). The limb lengthening protocol proposed in this study is the staged limb lengthening protocol, which involves simultaneous bilateral lengthening of the tibia and femur with EF in four surgical stages, as described by Peretti et al. (11). Our study aims to analyze the results and complications of our lengthening protocol in patients with achondroplasia. In addition, the present work aims to analyze any significant differences between tibial and femoral lengthening by comparing length gain, consolidation time, time with EF, and associated complications.

## 2. Materials and methods

A retrospective analysis was performed on 33 patients with achondroplasia undergoing simultaneous bilateral tibia and femur lengthening in four surgical stages, as described by Peretti et al. (11). All patients (21 females and 12 males) were operated on by the same orthopedic surgeon from 2017 to 2021. The diagnosis of achondroplasia had already been made by specialists at an early age for all patients who came to our institution to undergo the lower limb lengthening procedure. An initial interview was conducted to assess the patient's physical condition and any comorbidities, explain the protocol, describe the principle of operation of an external fixator and its management at home, elucidate clinical goals and possible complications, estimate the duration of treatment, frequency of follow-up visits, and the importance of physiotherapy and psychological support. Following written informed consent, all these patients underwent surgery. Forty-six lengthening procedures were performed, with a total of 92 bone segments lengthened (56 tibias and 36 femurs). The mean age of patients at first surgery was 8.73 years (range 6–15). At the time of data analysis, five patients had completed the whole lengthening protocol, fourteen patients completed the 3° step, seven patients the 2°, and seven the 1° step. Table 1 describes patients and bone length characteristics. Clinical details and antero-posterior and lateral radiographs were obtained from archived medical records, the hospital computer service, and the image archiving and communication system. The parameters analyzed were: the duration of lengthening with EF and its removal, the lengthening device, the amount of lengthening achieved for each bone segment (through analysis of radiographs), the presence of complications and events that influenced the outcome, and the healing index (HI), expressed as days of EF application per centimeter of lengthening of each bone (12).

**Table 1:** Patients and bone length characteristics.

Patients	*n* = 33
Mean age at 1° step (years)	8.73
Sex	Female 21
	Male 12
Step at time of data analysis	7 (1° step)
	7 (2° step)
	14 (3° step)
	5 (4° step)

### 2.1. Surgical procedure and follow-up

The procedure of limb lengthening was performed in four surgical steps as described by Peretti et al. (11). All patients underwent simultaneous bilateral transverse lengthening of the affected bones; the first phase of our protocol involves the tibiae, the second the femurs, the third the tibiae, and the fourth the femurs. Patients could access the different phases approximately 1 year after EF removal. A ring fixator with a 2-ring structure was used for tibial lengthening, an osteotomy was performed at the level of the distal 1/3 of the fibula with multiple drill holes through an anterior approach and completed with an osteotome. The proximal ring was applied parallel to the knee joint, and the distal ring parallel to the ankle joint. The second surgical step consisted of another osteotomy of the tibia at its proximal 1/3 with the same technique. The periosteum was then carefully closed. The rings were connected to each other through adjustable bars. A single-sided pediatric limb reconstruction system was used for femoral lengthening; in this case, a single osteotomy of the distal diaphyseal-metaphyseal junction was performed. After surgery, there was an initial latency phase, and distraction was started after the fourth postoperative day in the ward, achieving a daily increase of 1 mm. The average length of hospitalization was about 5–7 days for each surgery. During hospitalization, special attention was paid to physiotherapy; regular range of motion (ROM) physiotherapy was provided for adjacent joints, particularly the knee and hip. The patient was encouraged to walk with the help of a walker to reduce osteopenia from immobilization, improve circulation, and maintain muscle mass.

**Figure 1:**
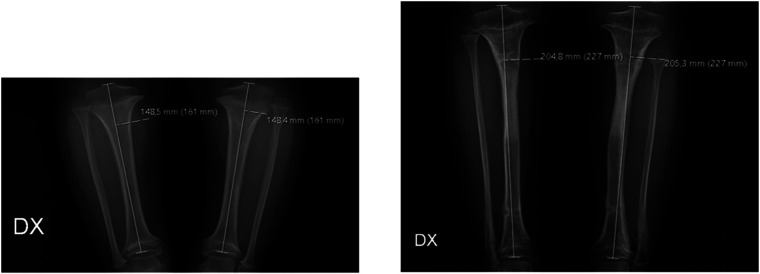
Radiographic measurements of the tibia before surgery (left image) and after removal of the external fixators (right image).

**Figure 2:**
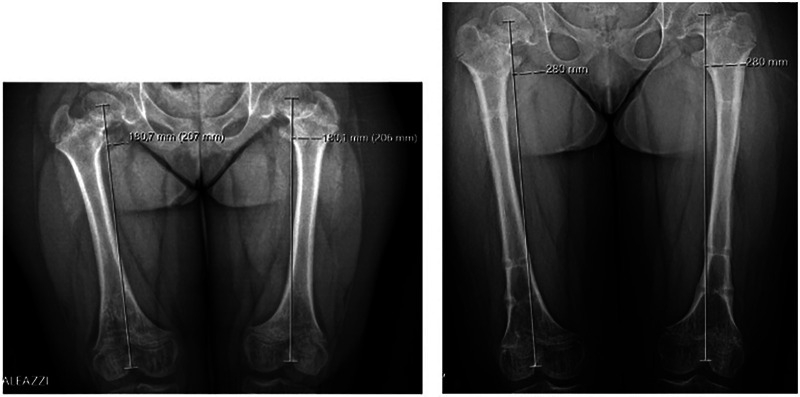
Radiographic measurements of the femur before surgery (left image) and after removal of external fixators (right image).

The first follow-up visit was scheduled ten days after hospital discharge, then patients were monitored monthly, and during those visits, all joints were clinically and radiologically examined. When the planned lengthening was achieved, the distraction phase was stopped, and the consolidation phase began to allow the regenerated bone to consolidate. The EF was removed only after three cortices of the regenerated bone in the antero-posterior and lateral radiographs were observed. In specific cases, the bone segments were protected for 4–6 weeks with patella tendon support casts for the legs and prophylactic titanium elastic nails (TENs) for the femur (13). Radiographic measurements were performed using the Picture Archiving and Communication System (PACS). x-rays were performed using a radiopaque calibration object, a sphere with a known diameter of 20 mm. Acquired length (cm) was defined as the difference in the length of the long bone measured two times, immediately before and after lengthening. The tibial length was measured from the most proximal part of the tibial eminence to the midpoint of the tibial plafond (14) (Figure 1). Femur length was measured from the most proximal part of the femoral head to the most distal part of the femoral condyle (Figure 2). Both absolute and percent elongation from initial length were calculated together with HI.

### 2.2. Statistical analysis

Statistical analyses were performed using R software v4.1.3 (R core Team, Vienna, Austria). The distribution of continuous variables was tested with the Shapiro–Wilk test; based on the test results, parametric and nonparametric tests were applied to compare subgroups (t-student or Mann Whitney). Correlation tests were performed according to Pearson's and Spearman's methods. Differences between the proportions of categorical variables were assessed by Fisher's exact test or chi-square test for trend, in case of multiple ordered categories. Logistic regression models, selected according to Akaike's information criterion (AIC), were run to assess the influence of different variables on the occurrence of complications. *P* values less than 0.05 were considered statistically significant. *P* values less than 0.1 were considered as significance trends.

## 3. Results

### 3.1. Tibial lengthening

**Figure 3:**
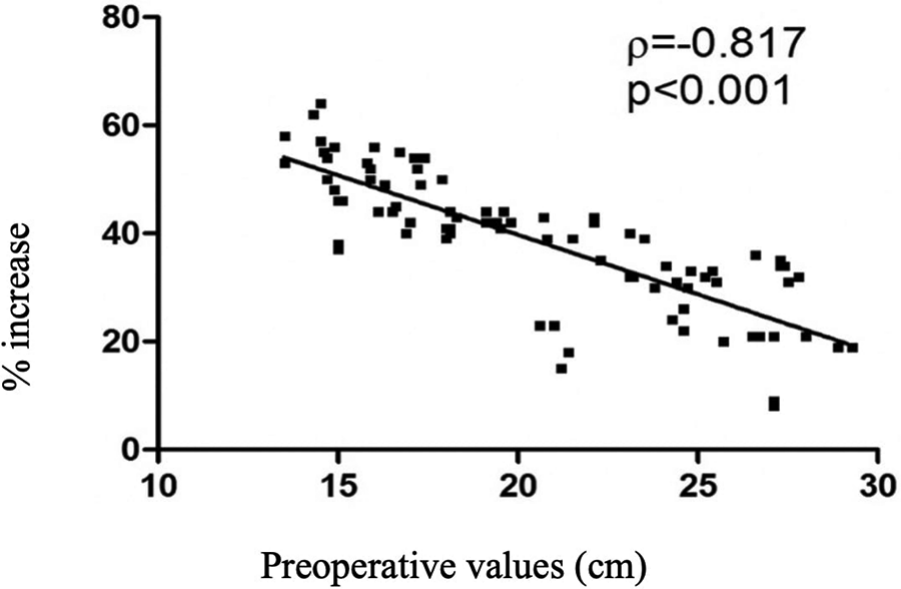
Scatter plot showing the negative correlation between preoperative bone size and percentage of increase.

**Figure 4:**
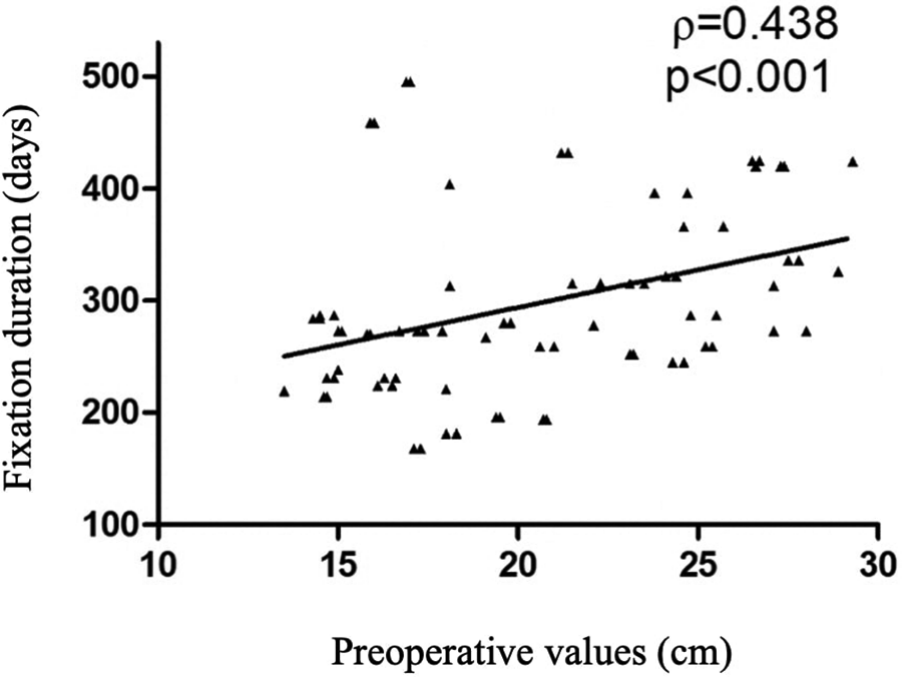
Scatter plot relating fixation duration and preoperative values.

**Figure 5:**
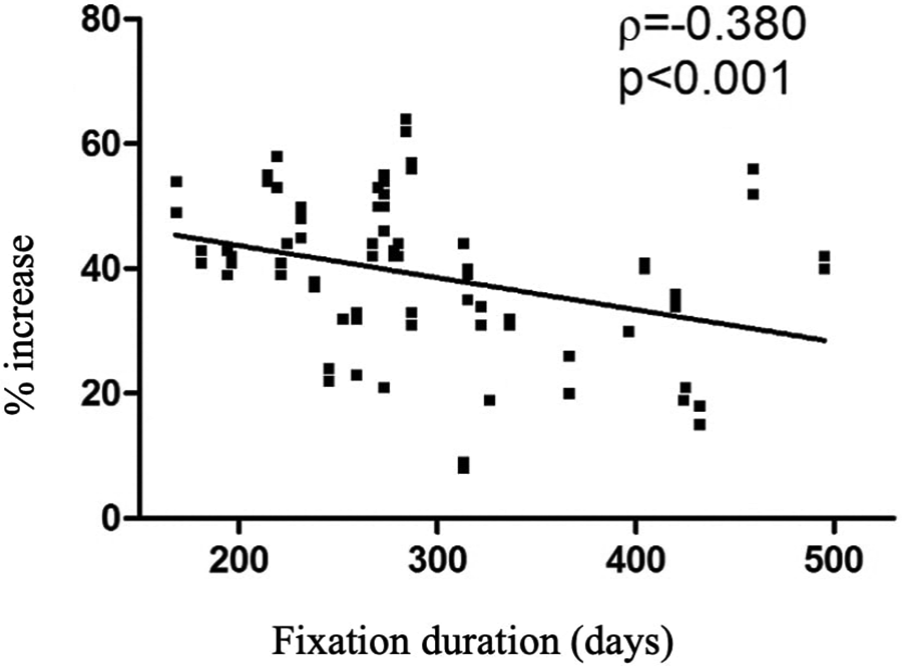
Scatter plot relating the percentages of increase and the duration of fixation.

The average tibial gain was 7.9 cm (range 5.1–9.7 cm). Planned lengthening of 30% or more was achieved in 93% of patients. The greatest gains were achieved in the first step of tibial lengthening compared with the second step (*p* < 0.001). No significant differences were reported regarding patients' gender. A negative correlation between the percentage of increase and the preoperative value was found (*p* < 0.001, *r* = −0.817), meaning that the smaller the initial length, the greater the gains obtained (Figure 3). The mean duration of EF was 295 days (9.7 months) and the mean healing index for the tibia was 38 days/cm. Significant differences in fixation duration by step were reported (1st vs. 3rd, *p* = 0.005) with no significant differences between males and females (*p* = 0.582). In addition, significant correlations emerged between fixation duration and preoperative bone length (*p* < 0.001, *r* = 0.438) and augmentation percentage (*p* < 0.001, *r* = 0.380) (Figures 4, 5). The positive association with preoperative bone length indicates a longer fixation time with greater length, while the negative association with augmentation percentage indicates a lower augmentation percentage when the duration is longer.

### 3.2. Femoral lengthening

**Figure 6:**
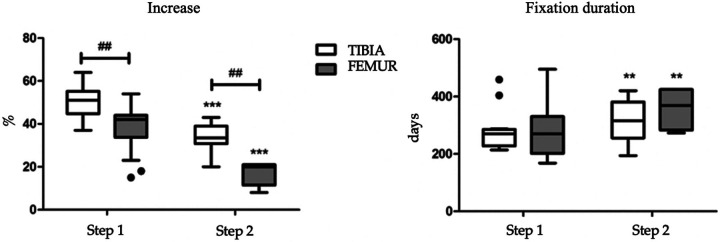
Comparison of tibia and femur with respect to percentage increase (left) and duration of fixation (right).

At the end of the femoral lengthening procedure, the average lengthening achieved was 6.9 cm (range 2.1–9.5 cm). Planned lengthening of 30% or more was achieved in 67% of patients. Greater gains were obtained in the first stretching procedure compared to the second step. No significant differences were reported regarding patients' gender. The average duration of fixation in the femur was 302 days (9.9 months) with an average healing index of 44 days/cm. Comparing femoral and tibial lengthening (Figure 6), the tibiae achieved better results than femurs (*p* = 0.005).

### 3.3. Complications

**Figure 7:**
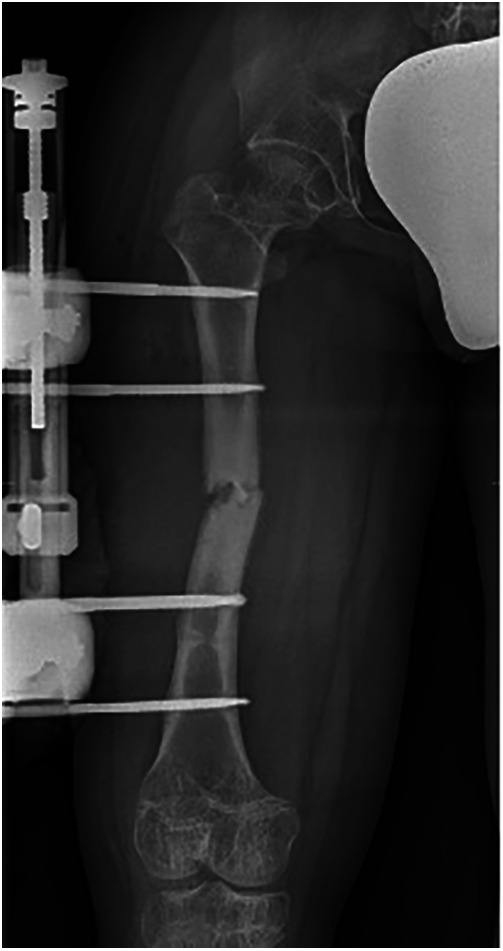
Postoperative radiograph after placement of a uniaxial external fixator following diaphyseal fracture of the right femur.

**Figure 8:**
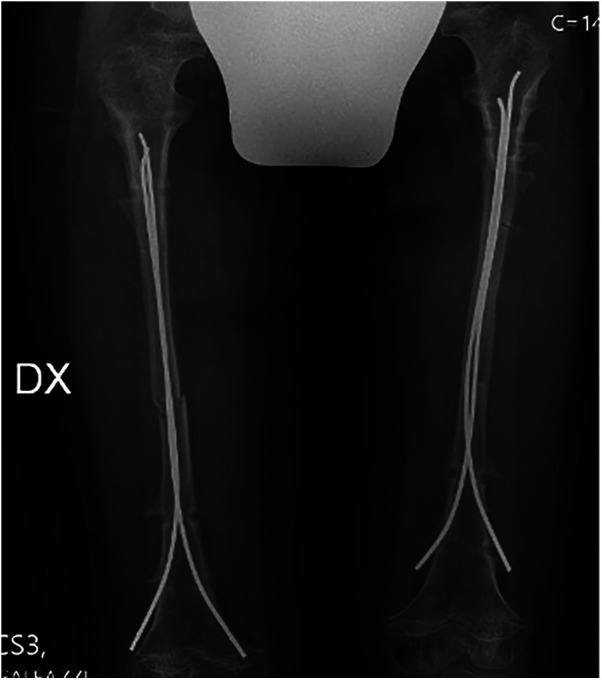
Postoperative radiograph after TENs placement following bilateral femur fracture.

We defined the complications as “all adverse and unexpected conditions that altered the plan of care”, as defined by Ilizarov (15). Superficial skin infections were not included, which, although they occurred in most patients, were mild and rapidly healed. There was a total of 19 complications for 92 segments (20.7%, 9 tibial and 10 femoral), and are summarized in Table 2. The most common complication, which occurred in 7 patients, was premature consolidation. This complication occurred in 5 cases in the fibula and 2 cases in the femur. Those patients were treated with revision corticotomy. All patients who underwent premature consolidation achieved the expected lengthening. One patient underwent corticotomy for the opposite reason: delayed union of the regenerate in the femur, bilaterally; after a conservative approach, corticotomy resolved the problem and the patient achieved a 41% percent gain of original length. In two patients, a fracture of the bone regenerate occurred after EF removal due to an accidental fall; in one case the fracture involved both femurs, in the other only the right one. The two patients were differently treated: one subject underwent repositioning of the EF, while the other subject with a bilateral fracture was treated with titanium elastic nails (TENs) (Figures 7, 8). Only one patient had an infection during the femoral lengthening process. The patient manifested fever and increased inflammatory indices. The patient tested positive for methicillin-sensitive S. aureus and was successfully treated with antibiotic therapy without removing the EF. This adverse event is classifiable as a grade II infection according to Saleh Scott and Checketts–Otterbuns (16). We had 1 patient with a neurologic complication, a popliteal sciatic nerve stupor on day 1 after surgery. The patient was returned to the operating room for removal of a Kirschner wire. The symptoms resolved completely in the following days.

**Table 2:** Complications.

Complication	Site	Laterality	Treatment
Premature consolidation	Fibula (5)	Monolateral (4)	Corticotomy
	Femur (2)	Bilateral (3)	
Delayed consolidation	Femur (1)	Bilateral	Corticotomy
Fracture after removal of EF	Femur (2)	Monolateral (1)	Repositioning of EF
		Bilateral (1)	Nailing
Poor quality of the bone regenerate at the end of treatment	Tibia (1)	Bilateral	Prophylactic titanium elastic nailing (TEN)
Deep infection with elevation of inflammatory indices	Femur (1)	Monolateral	Antibiotic treatment
Stupor of the external popliteal sciatic nerve	Leg (1)	Monolateral	Replacement of K-wire

There were no significant differences between femur and tibia in terms of complications. Comparing the first steps (1st and 2nd) and second steps (3rd and 4th), more complications occurred in the first steps (17/54) than in the second steps (2/36) with a significant difference between the proportions (*p* = 0.002). Considering gender, age, step, bone segment, fixation duration (weeks) we used multiple logistic regression analysis and the only variables influencing complications were: step (*p* = 0.002) and fixation duration (trend, *p* = 0.061). This trend suggests that each week of EF is associated with a 4.7% increased risk of developing complications.

## 4. Discussion

This study shows that our four-stage limb lengthening protocol was able to perform an average gain of 7.9 cm with a mean duration of EF of 295 days for the tibias and an average gain of 6.9 cm with an average duration of EF 302 days for the femurs. In addition, this protocol reports a decent complication rate, compared to the available literature. Nineteen complications were reported for 92 segments (20.7%). The present study also underlies which factors could modify the complication rate. The only variables influencing complications were: step (*p* = 0.002) and fixation duration (trend, *p* = 0.061).

The literature shows that tibial and femoral lengthening represents a stressful orthopedic procedure associated with multiple adverse events. From the studies conducted by Chilbule et al. (17) and Burghardt et al. (18), the complications were 33 out of 36 lengthened segments (92%), 28 out of 28 lengthened segments (100%), respectively. In contrast, in the study by Venkatesh et al. (19), all 20 patients reported temporary joint stiffness and fractures of bone regenerate occurred in 15% of cases. Extensive stretching can also lead to excessive pressure on the open growth cartilage, resulting in growth inhibition: this was demonstrated in a study by Song et al. (20), which found premature closure of the physis after lower limb lengthening in more than 50 percent of patients with achondroplasia. Numerous stretching protocols are tested in achondroplasia, but there still needs to be more specific guidelines. In our study, moderate, parallel, bilateral lower limb lengthening was adopted and fractionated into four surgical steps, as proposed by Peretti et al. (11). We observed a lower complication rate using this method than in the literature. More specifically, out of a total of 46 lengthening procedures, 38 achieved the intended goal. The average bone length gain was 7.9 cm for the tibia and 6.9 cm for the femur, with an overall average height gain at the end of treatment of 29.6 cm. We hypothesize that splitting the lengthening into four times reduces the trauma on the soft tissues and joints. We obtained better results during the first two steps and in the tibia than in the femur; this latter aspect was also shown by Park et al. (21) in bilateral parallel lengthening but performed in only two steps (1° step tibiae, 2° step femurs). Consistent with the Griffith et al. study, we did not find a higher rate of adverse events in patients undergoing successive lengthening of the same bone; on the contrary, we saw a significant decrease in the frequency of complications (22). This decreased rate is probably due to an adequate recovery time between procedures, waiting at least 1 year after EF removal to proceed with the following procedure, which translates into a minimum wait of 2 years between the start of one lengthening and the next. In support of this claim, Tjernström et al. suggested that the time required for bone remodeling after a lengthening is at least 1 year (23). A more detailed comparison of the experience of other authors is presented in Table 3.

**Table 3:** The experience of other authors in limb lengthening.

Author, Journal	Year	Surgical protocol	Patients	Results	Complications
Shabtai, L. *Children* (**24****)**	2021	External Fixation. Two steps. I: tibiae and femurs II: tibiae and femurs	50	Lenghtening: • Mean tibiae lenghtening: 5.2 cm, mean HI tibiae: 1.4 months/cm • Mean femurs lenghtening: 7.2 cm, mean HI femurs: 1 month/cm Mean duration of external fixation: • 6.7 months (range 4.4–10.5 months)	76% complication rate, 45% of these required additional surgery
Paley, D. *Children* **(****25****)**	2021	External fixation or intramedullary nail. Single step. Tibiae or femurs (14 patients) Tibiae and femurs simultaneously (64 patients)	75	Lenghtening: • Mean height gain 27 cm in achondroplasia • Mean height gain 17 cm for hypoachondroplasia Mean duration of external fixation: NA	1% permanent sequelae
Dossanov B. *Nature, Scientific Reports* **(****26****)**	2021	External fixation. Single step: tibiae (8 patients) or femurs (4 patients)	12	Lenghtening: • Regenerate lenght: 8.5 ± 0.6 cm • % increase: 53% ± 5% Mean duration of external fixation: • 83.8 ± 3.7 days	50% complication rate
Leiva-Gea A. *Arch Ortho Traum Surg* **(****27****)**	2020	External fixation. Two steps. I: simultaneous bilateral lengthening of the femur and tibia II: humeral lengthening	21	Lenghtening: • Tibiae + Femurs: 14.43 ± 1.41 cm Mean duration of external fixation: • 261.12 ± 50.11 days	45% complication rate
Ko, K. *Clin Orthop Surg* **(****28****)**	2019	External fixation. Three steps I: controlateral tibia and femur II: controlateral tibia and femur III: humeri	15	Lenghtening: • Mean tibiae lenghtening: 8.5 cm, mean HI tibiae: 29.0 days/cm • Mean femurs lenghtening: 8.3 cm, mean HI femurs: 29.6 days/cm Mean duration of external fixation: NA	85% complication rate
Chilbule SK. *Indian J Orthop* **(****17****)**	2016	External fixation. Two steps. I: bilateral tibiae II: bilateral femurs III: bilateral humeri	9	Lenghtening: • Mean tibiae lenghtening: 15.4 cm, mean HI tibiae: 25.7 days/cm • Mean femurs lenghtening: 9.9 cm, mean HI femurs: 25.6 days/cm Mean duration of external fixation: NA	90% complication rate
Donaldson J. *J Orthop* **(****29****)**	2015	External fixation. Two steps. I: controlateral tibia and femur II: controlateral tibia and femur	10	Lenghtening: • Height gain: 20.5 cm Mean duration of external fixation: NA	70% complication rate
Park KW. *Yonsei Med J* **(****21****)**	2015	External fixation. Two steps. I: bilateral tibiae II: bilateral femurs	28	Lenghtening: • Tibiae mean lenghtening: 9.8 cm • Femurs mean lenghtening: 8.4 cm Mean duration of external fixation: NA	95% complication rate
Burghardt R. *J Orthop* **(****18****)**	2015	External fixation. Single step: Tibiae	14	Lenghtening: • Tibiae mean lenghtening: 13.5 cm • Mean Healing index: 0.7 months/cm Mean duration of external fixation: • 8.8 months	100% superficial infections 20 peroneal nerve damage (reversible) 3 premature consolidation 5 rigidity
Kocaoglu M. *Acta Orthop Traumatol Turc* **(****30****)**	2014	External fixation. Single step simultaneous bilateral lengthening of the femur and tibia	22	Lenghtening: • Mean tibiae lenghtening: 6.64 cm, mean HI tibiae: 34.3 days/cm • Mean femurs lenghtening: 7.07 cm, mean HI femurs: 31.2 giorni/cm • Mean height gain: 16.9 cm Mean duration of external fixation: NA	82% complication rate
Kim SJ. *Clin Orthop Relat Res* **(****31****)**	2012	External fixation. Two steps. I: bilateral tibiae II: bilateral femurs	22	Lenghtening: • Mean tibiae lenghtening: 9.1 cm, mean HI tibiae: 35 days/cm • Mean femurs lenghtening: 10.2 cm, mean HI femurs: 34 days/cm Mean duration of external fixation: NA	100% complication rate
Venkatesh K. *J Bone Joint Surg* **(****19****)**	2009	External fixation. Protocol not specified Femural lenghtening	20	Lenghtening: • Femurs mean lenghtening: 9.2 cm (39.3%) Mean duration of fixation period: • 10.8 months	100% rigidity 15% fractures
Vaidya SV. *J Pediatr Orthop* **(****32****)**	2006	External fixation. Protocol not specified Tibial lenghtening	24	Lenghtening: • Tibiae mean lenghtening: 6.84 cm, mean HI tibiae: 26.06 /cm Mean duration of external fixation: NA	97% complication rate

Our study also demonstrated that each week of EF placement is associated with a 4.7% increase in the risk of developing complications. Therefore, the duration of EF treatment should be sufficient to ensure satisfactory lengthening and good-quality bone regenerate. At the same time, external fixation should not be excessively prolonged to avoid further complications. Thus, in cases where the quality of the bone regenerate is still not optimal after a long period of fixation, removal of the EF and preventive treatment with titanium elastic nails (TENs) is recommended to prevent the fracture risk. This procedure is the “lengthening then rodding”, which has already been described in the literature and tested in one of our patients (13).

Our study has several strengths: it ranks third in terms of sample size among scientific studies of the last two decades on lower limb lengthening with EF, preceded by a study by Paley (25) (75 patients, including 66 patients with achondroplasia) and a study by Shabtai et al. (24) (50 patients). Moreover, the same surgeon performed all lengthening procedures, thus zeroing interoperator variability. The data we collected enrich the current literature, which needs to include recent results regarding fractional lengthening in four surgical steps performed in pediatric patients 6 years of age and older. Finally, the results of the present study also offer some insights for further studies, such as the preventive treatment of regenerated fractures with TENs. Our study has some limitations that need to be considered. First, it is a retrospective, noncomparative study conducted in a single center, with no direct comparison between extensive and moderate lengthening and different surgical protocols. Second, this retrospective analysis did not include quality-of-life questionnaires or patient-reported outcome measures (PROMs) at the beginning and end of treatment, which is essential to integrate clinical outcomes and understand the real impact on the patient's daily life. However, Kim et al. (31) showed that limb lengthening improves the quality of life of patients with achondroplasia despite the high rate of complications. In addition, questionnaire scores improve significantly if humerus lengthening is also performed (8), a procedure that only three of our patients have undergone so far. Third, other fixation methods are now available for limb lengthening, such as intramedullary nails, which still need to be tested in our center and might be interesting to compare with EF results. Such devices are commonly used in limb lengthening with underlying etiologies other than dwarfism of achondroplasia. Indeed, they have as a limitation the preoperative bone length that must be at least 17.5 cm in order to lengthen the limb by 5 cm, and this requirement is not always met in the child with achondroplasia. However, in the aforementioned Paley study, the intramedullary nail is identified as a promising alternative to EF, as it could be associated with fewer complications and less psychological stress for the patient (25).

In our opinion, the future challenge of surgery will be finding the correct treatment combined with the latest pharmacological therapy (33). Finally, as already expressed in the literature, we reiterate the importance of a multidisciplinary approach to the patient with achondroplasia. Pediatricians, geneticists, pulmonologists, neurologists, endocrinologists, and rheumatologists should be involved and collaborate with the figure of the orthopedic surgeon (34). Furthermore, we reiterate the importance of close collaboration with physical therapists, ideally specialized in these highly specific procedures. Constant monitoring and efforts to maintain the ROM in adjacent joints, the assessment of motor patterns, and the preservation of muscle trophism are fundamental pillars of this therapeutic synergy. In addition, our patients and their families are always counseled with psychological support throughout the lengthening process to help them cope with the long journey ahead and possible complications.

## 5. Conclusion

Our study suggests that bilateral parallel lower limb lengthening, fractionated into four surgical steps and undertaken at pediatric age, could be a viable technique in patients with achondroplasia. Based on our experience, we suggest that limb lengthening should be started at the pediatric age of 6–8 years. The future challenge of surgery will be finding the ideal treatment combination with drug therapy. A multidisciplinary approach to the patient with achondroplasia, together with psychological support, remains recommended for all patients who undergo this path.

## Data Availability

The original contributions presented in the study are included in the article/Supplementary Material, further inquiries can be directed to the corresponding author.
